# Validity of the Early Years Check-In (EYCI) in a Cross-Sectional Sample of Families

**DOI:** 10.3389/fped.2020.00157

**Published:** 2020-04-24

**Authors:** Heather Clark, Kalpana Nair, Scott Veldhuizen, Wenonah Campbell, Lisa Rivard, M. Christine Rodriguez, John Cairney

**Affiliations:** ^1^Faculty of Kinesiology and Physical Education, University of Toronto, Toronto, ON, Canada; ^2^Faculty of Health Sciences, School of Nursing, McMaster University, Hamilton, ON, Canada; ^3^Faculty of Health Sciences, School of Rehabilitation Science, Institute of Applied Health Sciences, McMaster University, Hamilton, ON, Canada; ^4^School of Human Movement and Nutrition Sciences, University of Queensland, St Lucia, QLD, Australia

**Keywords:** surveillance, screening, developmental delay, parental concerns, early years

## Abstract

**Background:** The objective of the present study was to develop and test the validity of the Early Years Check-In (EYCI), a new tool that measures parent and educator concerns regarding children's development. The study examined the EYCI's agreement with 3rd edition of the Bayley Scales of Infant and Toddler Development (BSID-III) an established measure of child development. Two possible thresholds were explored: one to identify children with a probable delay, and another to identify children at the borderline functioning threshold.

**Methods:** Parents of children aged 18 to 42 months were recruited from childcare settings across Ontario, Canada. The study proceeded in two phases. Phase I, intended to pilot the measure, included 49 children. Phase II, a test of the validity of the final version, included 199 children. Parents and educators completed the EYCI for the child, while a blinded assessor completed the BSID-III.

**Results:** The EYCI demonstrated good sensitivity and specificity (86 and 82%, respectively) as a parent-completed tool that identifies children with a probable delay. However, the positive predictive value (15%) suggests the EYCI is likely to over identify children. When identifying children who demonstrated borderline delay, the EYCI demonstrated good sensitivity (80%) but poor specificity (49%). Results from educator-completed EYCIs were poor for both probable and borderline delay.

**Conclusions:** While further research is required, the EYCI shows promise as a parent-completed tool, particularly to identify more-severe cases of delay. Results with educators were poor overall. Future research investigating accuracy of educators in different types of early childcare centres is needed.

## Introduction

Identification of delay and vulnerability during the early years is an important first step to connecting families to services and supports (1–3). The early years represents a time of neuroplasticity and investing in addressing developmental problems can have a significant impact on later development (4–6). Despite interest in identifying developmental vulnerability and problems in early childhood, concerns remain about the coverage of current surveillance and screening efforts, which largely occur within primary care, and the number of children who start school with a delay or low readiness for school, especially among low-income groups (7, 8). At present, there is a lack of tools and research that align with current practice guidelines regarding developmental surveillance and screening.

Current guidelines in both Canada (9) and the United States (10, 11) highlight the importance of attending to parental concerns as part of regular monitoring and surveillance efforts in primary care, such as well-baby visits. Universal screening is not recommended. Instead, when parental concerns are present, then screening might be necessary (9–13). Research examining how parents reflect on their concerns suggests that an informal question about concerns, or even a single question about concerns is insufficient (14). An alternative approach is to use a tool to elicit parental concerns.

When examining tools and approaches to developmental surveillance and screening, it is important to consider both the accuracy of tests, and the consequences of applying the tests in contexts where prevalence is low (15). Regarding test accuracy, it is recommended that tests have a sensitivity (the ability to detect true cases) of 80% and specificity (the ability to exclude non-cases) of 70% (16). When prevalence of a disorder is low, the number of false positives is high and the positive predictive value (PPV), the proportion of true positive relative to false positives, is low. The negative predictive value (NPV), which is the proportion of people who score negative on the test that do not have this disorder, is also low. The low PPV is an important issue for assessing delay because estimated prevalence of delay among children and youth range from about 4.1% to about 13% (16–18). Therefore, even an instrument that meets sensitivity and specificity guidelines is still likely to have low PPV and by extension, a large proportion of false positives. There are no set guidelines for PPV or NPV in surveillance and screening. The cost and impact of a false positive or a false negative should be considered when determining acceptable PPF and NPV values. If the cost of a false positive is high, either in financial resources related to referrals and further assessment, or in terms stress for families, than a low PPV is problematic. Therefore, any universal developmental surveillance approach should consider how to keep the risks associated with a false positive low.

The Parents' Evaluation of Developmental Status (PEDS) (19) is a well-known measure of parental concerns. The PEDS includes 8 items asking parents whether they have any concerns (no, yes, a little) about cognitive functioning, language, motor skills, or social functioning. Two additional items ask parents to list their concerns. The PEDS has been employed for both developmental surveillance, to elicit parental concerns in primary care practice, and as a developmental screening tool. However, research on the psychometric properties of the PEDS has reported variable results. Sices et al. (20) found good sensitivity and specificity of the PEDS in a small (*n* = 60) sample of young children aged 9 to 31 months attending primary care. Glascoe (21) reported similar results with a sample (*n* = 295) of children aged age 4.5 and older, however the sensitivity and specificity for children under age 4.5 were 0.68 and 0.66, respectively. Limbos and Joyce (22) reported a sensitivity and specificity of 0.73 and 0.68 in a 334 children aged 12–60 months. PPV values for the PEDS have varied from 19 (22) to 50% (20), while other studies have reported values between 20 and 30% (21, 23). The variability in results of the PEDS is not unique, but rather the rule in terms of developmental screening tools. For example, results of another commonly used screening tool, the Ages and Stages Questionnaire (ASQ) (24), has wide variation in results (22, 25–29).

Another important consideration in developmental surveillance is how to attain maximum coverage of the population. Primary care contact in the early years, such as regular check-ups and immunization visits with physicians, are important but may be insufficient for regular monitoring and surveillance. While many jurisdictions offer regular check-ups during the first year to 18 months, these become less frequent after 18 months (30), just as developmental issues become more apparent (25, 31, 32). Also, a number of challenges have been identified with well-baby visits including: lack of time, lack of training, and resistance to changing practice (33). Research examining developmental surveillance and screening practice has reported large variability in whether surveillance or screening occurred as well as the methods used. In the U.S., the proportion of families with young children aged 9 to 35 months who received developmental surveillance from a health professional in the last year varied across States from about 19 to 60%. Rates of developmental screening were only slightly lower from 17 to 59% (34). In terms of methods used, recommendations vary by region. In Ontario, the Nipissing Developmental Screen and Rourke Baby Record be used in conversations with parents (35). These challenges highlight the need for a comprehensive model of population monitoring and surveillance that involves settings and professionals beyond primary care.

Incorporating monitoring and surveillance into early childcare settings can complement existing practices in primary care. There are many features of early childcare settings that can facilitate the identification of children who show signs of delay or vulnerabilities. Registered Early Childhood Educators (RECEs) are trained in child development, see families on a regular basis, have multiple opportunities to observe a child, and can offer connections to other organizations and services when there is an identified need (36, 37). Regular surveillance and screening already occur in school-aged children and it is accepted that using teacher-reported instruments in schools can be an effective method for identifying school-aged children who show signs of delay or developmental vulnerability (38–40). In contrast, developmental surveillance in early childcare settings is an area that has received little attention in the research literature. This persists despite concerns regarding under-identification of delay at this age (2, 3, 18). Recent studies have supported the feasibility of developmental screening in childcare centres, with providers believing it is an important part of their role (41, 42). Unfortunately, these studies focused on screening rather than surveillance.

The few studies that have been conducted in early childcare settings show promise. Dereu et al. (43) found an early educator completed tool showed better sensitivity and specificity than parent-reported instruments in detecting autism in children ages 3 to 39 months. Other research has reported that with regular assessment by early childhood educators, earlier identification of children with autism can occur (44, 45). This work, however, is limited as it has focused solely on autism. Exploring the ability of tools to detect other kinds of developmental problems and delays in this setting is required.

Only a small percentage of children attend licensed childcare (LCC) facilities. It is difficult to get an exact estimate as both participation in programs, and how programs are defined varies by region. In Canada about 20–38% of children under 4 attend a daycare centre (46). This number does not distinguish between licensed and unlicensed centres, so represents an overestimate of licensed childcare attendance. Over 50% of young children in the United States have been reported in centre-based care, but the degree that these are monitored and licensed varies widely by State (46–48). While licensed childcare offers potential for developmental surveillance, it is not sufficient to provide wide coverage of young children. There are however, a number of community-based family-focused centres that offer programs for parents and their families. These centres offer a way to increase the reach of surveillance efforts beyond LCC settings. In Ontario, Canada, these include Early Ontario Child and Family Centres (EarlyON), which offer programming for children ages 0-6 years and their parents. These centres are staffed with professionals, such as RECEs, and act as a community hub for connections to other organizations, including public health and other agencies that offer secondary and/or tertiary services (49).

## Development and Rationale Of The Early Years Check-In (Eyci) Tool

We developed the Early Years Check-in (EYCI) for use by parents and early years professionals (i.e., early educators in early childcare settings). The EYCI was developed with input from parents, early educators, and experts in child development, psychometrics, and health measures scale development. The tool was designed for parents and ECE's of children ages 18 months to 6 years; 18 months was set as the lower age band due to difficulties detecting delay at earlier ages (25, 31, 32). We wanted to ensure coverage across 4 developmental domains (social-emotional; motor; language; and cognitive). We started with focus groups with parents and educators to learn what areas parents and educators think about development, and factors that would facilitate or discourage use. Our results supported the use of 11 questions, with 10 relating to a different area of potential concern and one overall or “global concern” question. The domains of the EYCI largely align with the PEDS, but with two additional areas that capture concerns regarding emotions and overall concerns. Results from focus groups also indicated that for the tool to be used, it needed to be easy to understand and quick to complete. Parents also preferred a visual analogue scale (VAS) to express their concern rather than boxes or numbers (another critical difference between the EYCI and the PEDS). Both parents and educators liked the blue color gradient used in the VAS as it felt more neutral than other colors (particularly, red, orange, or yellow). Once we developed the EYCI questions, we completed cognitive interviews to ensure questions we understood by parents and educators. Further detail regarding the tools development has been reported elsewhere (13).

We conducted a psychometric examination of the EYCI in two phases. The goals of phase I were to: (1) evaluate the item functioning of the EYCI (e.g., response patterns and reliability), (2) measure initial agreement with a standardized instrument, and (3) understand parental concerns by measuring their associations between the EYCI with parent and child history and functioning. The goals of phase II were to: (1) conduct a more robust test of the agreement with the standardized instrument and (2) identify thresholds for detecting probable and borderline delays. For both phases, the EYCI was completed by both parents and educators. Agreement between parents and educators on the EYCI tool was assessed, as well as the agreement of each type of rater with the BSID-III. The EYCI was developed as a surveillance tool, to identify children that further actions such as screening might be beneficial. As such, when testing the EYCI we aimed to maximize sensitivity.

## Methods

### Participants

**Figure 1:**
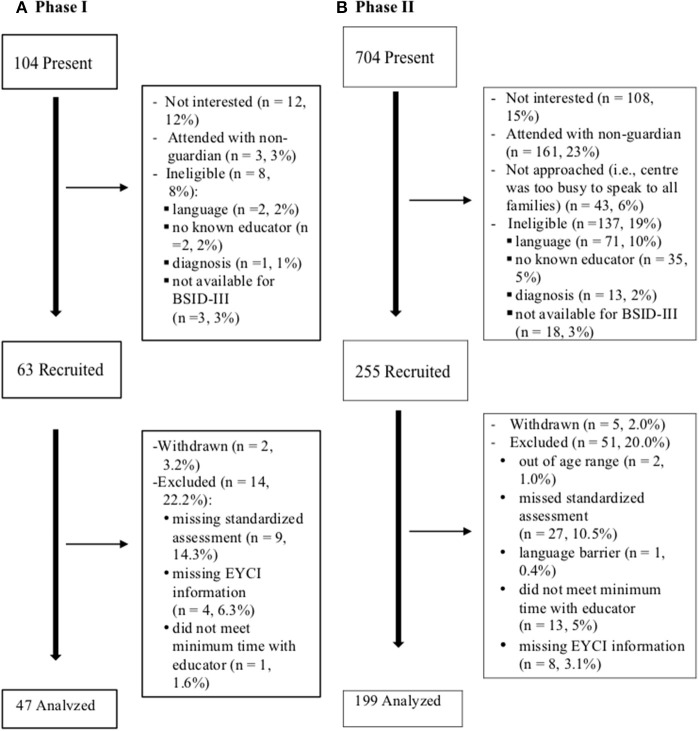
Study family recruitment and participation.

Both LCC and EarlyON centres were recruited in Ontario, Canada. As these centres are already mandated to support parents and children, and have established relationships with families, they were identified as optimal settings for this work. Phase I included 63 children recruited from a total of 104 children who attended 3 centres (2 OEYCs, and 1 LCC) in Hamilton, Ontario. Non-participating families were deemed ineligible (8%), declined participation (12%), or did not attend with a parent or guardian (3%). Phase II included 255 children recruited from a total of 704 children attending one of 28 centres across Ontario. Other families were ineligible (19%), did not attend with a guardian or parent (23%), or families declined participation (15%). Details of recruitment, and reasons for exclusion are outlined in Figure 1.

Inclusion criteria for families required ability to communicate in English, a child age between 18 and 42 months, and ability to attend a developmental assessment. The upper age-band was set based on the criterion measure employed in the study. We also only included families with a child that the educator had spent at least one hour with in the past 2 months (assessed via Educator report). We excluded all children when a parent reported a prior diagnosis of a developmental condition, such as cerebral palsy, muscular dystrophy, pervasive developmental disorder, an autism spectrum condition, attention deficit hyperactivity disorder, and developmental coordination disorder. This was done because there would naturally be concerns about children with existing diagnoses, which would artificially increase the measure's performance and because there is little clear benefit to identifying children with already-identified difficulties.

### Measures

#### Demographic Survey

Parents/guardians reported their age, marital status, age of parent at birth of their first child, their child's age, prematurity, and their own and their child's country of birth.

#### Access to Services

Parents completed a short survey regarding their child's current and past experiences with developmental services. The type of services included speech and language, physiotherapy, visual impairment, services related to behavioral issues, developmental delay, and other services.

#### Early Years Check-In (EYCI)

**Figure 2:**
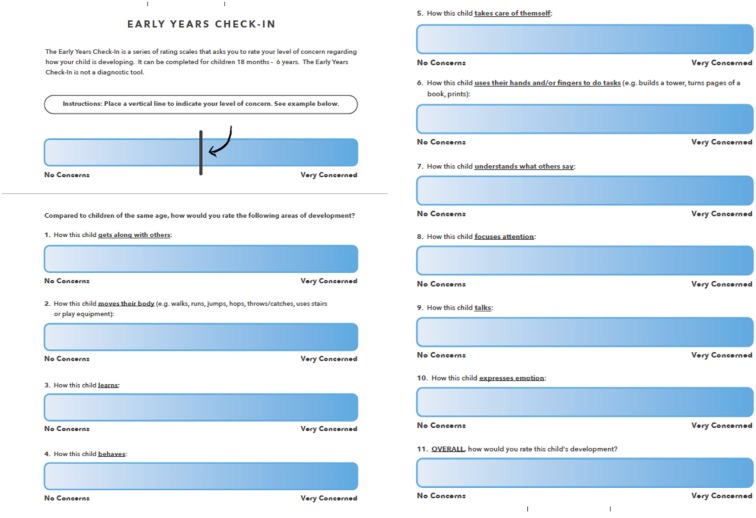
Early years check-in.

The EYCI assesses parental concerns across 11 items: 10 domain-specific items and one overall item that measures global concern. The full list of items is shown in Figure 2. Items are assessed using a 15 cm long VAS which is shaded in blue from left to right. A “No Concerns” anchor is on the far left end and “Very Concerned” is on the right end (see Figure 2). The EYCI was administered electronically and on paper. When used on paper, parents were asked to draw a vertical line on the scale; scoring took place by measuring from the left anchor to this line using a ruler. Two research team members measured each scale using the same ruler to ensure accuracy in scoring. When there was a discrepancy of under 0.5 cm the lower score was entered. When a discrepancy of greater than 0.5 cm was present a third person measured the item. Each time the third person measured the item, it agreed with one of the first two measures and the value that was consistent across 2 measures was entered. This occurred in ~1.3% of all completed EYCI's.

#### Developmental Functioning and Delay

The Bayley Scales of Infant and Toddler Development, 3rd edition (BSID-III) (50) was used to identify developmental functioning and delay. The BSID-III measures development in five domains: Cognitive, Language, Motor, Social-Emotional, and Adaptive. Cognitive, Language, and Motor are measured through a standardized assessment, and Social-Emotional and Adaptive are measured through a parent report questionnaire. For validation purposes, we used the lowest score on any domain to identify children with possible developmental issues.

### Procedure

The study received ethical approval from the Hamilton Integrated Research Ethics Board at McMaster University. Once a center agreed to take part, an information package that included the study process and consent forms were sent. The educator consent forms were reviewed, and interested educators provided informed, written consent. In all centres, study advertisements for families were displayed or distributed either electronically or on paper. Research staff attended centres and spoke to families, to share information and assess interest. Interested families were asked questions regarding their child's age, whether their child has ever received a diagnosis for developmental delay or disability, and the child's and caregivers' ability to communicate in English. Next they were asked if their child has spent time with an educator in the centre. If they did not know an educator at the centre they were excluded (see Figure 1).

Once families were recruited, they were invited to complete study materials on paper or electronically on a study tablet and an appointment for completing the BSID-III was scheduled within two weeks. Assessments were conducted by a registered psychometrist or trained research assistants who were blinded to the EYCI results. A list of participating children at each centre was compiled for educators to identify children with whom they were most familiar. If a family consented, but the child could not be matched with a participating educator, they were excluded from the study (see Figure 1). Educators were asked to complete the EYCI for the child within 2 weeks. If the educator needed more time, an extra week was given. This occurred at about 15% of centres.

### Analyses

We used alpha coefficient, a common estimate of internal consistency (51), and Spearman correlation to measure associations between parent and educator ratings and between individual EYCI items and BSID-III domains. Spearman was used due to the skewed nature of the data (51). To evaluate the ability of the measure to identify children with potential delay, we used receiver operating characteristic (ROC) curve analysis. The area under the ROC curve (AUC) is a widely-used measure of agreement, and can be interpreted as the probability that a randomly selected participant with a delay will have a higher score than a randomly selected participant without delay. Values from 0.5 to ~0.7 reflect poor accuracy, values of 0.7–0.9 reflect moderate accuracy, and values above 0.9 are highly accurate (52).

We sought to identify two thresholds for the EYCI: (1) a high concern threshold, where the advice of a health professional and further screening might be appropriate, and (2) an elevated concern threshold, that identified children who would benefit from closer monitoring and where actions *might* be appropriate. For the high concern threshold, we selected the “extremely low” threshold of the BSID-III (-2 SD. For the elevated threshold, we used the “borderline functioning” threshold of the BSID-III (-1.5 SD). We compared the highest score on any individual EYCI cut-point to the lowest level of functioning on any BSID-III domain.

## Results

### Sample Characteristics

The mean age in phase I was lower (*M* = 29.7 months, *SD* = 7.0) than phase II (*M* = 34.4 months, *SD* = 5.2). The majority of children in both samples were typically developing. In phase I, one child exhibited potential delays and six showed borderline delays. In the larger validation study (phase II), 7 children exhibited potential delays and 18 showed borderline delays. More detailed sample characteristics are described in Table 1. Twelve educators completed EYCIs for participating children across the centres in phase I, and 57 educators completed EYCIs for children in phase II.

**Table 1:** Description of samples.

	**Phase I**		**Phase II**	
	**M (SD)**	**Min, Max**	**M (SD)**	**Min, Max**
**Family demographics**				
Child age (in months)	28.0 (7.2)	18, 41	34.4 (5.2)	22, 42
Guardian age	33.8 (7.66)	26, 63	29.0 (6.84)	18, 42
Guardian age at birth of first child	26.2 (5.5)	15,41	29.6 (5.7)	16, 46
Number of children in household	2.2	1,5	2.1 (1.0)	1, 8
	***n***	**%**	***n***	**%**
**Guardian relationship to child**				
Biological mother	40	85.1	176	87.1
Biological father	4	8.5	21	10.4
Grandparent	3	6.4	0	0.0
Adoptive mother	0	0.0	2	1.0
Adoptive father	0	0.0	2	1.0
Non-birth mother in same sex couple	0	0.0	1	0.5
**Education**				
Less than secondary school	2	4.3	5	2.5
Secondary school	9	19.1	18	9.0
Some college or university	5	10.6	30	14.9
College diploma	10	21.3	48	23.9
Undergraduate or university degree (e.g., BA, BS., BFA)	16	34.0	65	32.3
Professional or graduate degree (e.g., JD, MA, PhD, MD, DDS)	5	10.6	35	17.4
**Country of birth**				
Canada	36	76.6	147	72.8
Other	11	23.4	54	26.7
**Marital status**				
Married/common law	43	91.5	185	91.6
Single (never married)	3	6.4	9	4.5
Separated/Divorced	1	2.1	8	4.0
**Child sex**				
Male	18	36.7	108	53.5
Female	31	62	94	46.5
**Premature birth**				
Not early	46	93.8	182	95.3
≥4 weeks prior to due date	3	6.2	9	4.7
**Child history of services**				
Never used services	41	89.1	170	84.2
History of services	5	10.6	29	14.4

^*^p < 0.05, ^**^p < 0.01.

### Phase I Results

The EYCI showed excellent internal consistency for parents (alpha = 0.95; 95% CI: 0.93-0.97) and educators (alpha = 0.93; 95% CI: 0.91-0.96). All items had item-total correlations above 0.3. Item distributions are presented in Table 2. All items were positively skewed, due to low levels of concern expressed by most participants. Parents used the full range of the VAS to rate their concerns as demonstrated by similar scores in the observed and possible minimum and maximum value. Educators had lower concerns than parents across all items. Across both parents and educators, two items were very low: *How this child moves*, and *How this child uses their hands*.

**Table 2:** Phase I Distribution of EYCI Items.

	**Parent**						**Educator**					
**EYCI Item**	**Mean**	**SD**	**Median**	**90th *p***	**Min**.	**Max**.	**Mean**	**SD**	**Median**	**90th *p***	**Min**.	**Max**.
Gets along	3.42	3.44	2.8	8.6	0	13.8	2.44	2.41	1.8	6.3	0.2	9.2
Moves	1.63	2.15	0.7	4.5	0	8.8	0.86	1.19	0.5	1.8	0.1	7.3
Learns	2.73	3.39	1.6	7.5	0	13.2	2.02	2.46	0.7	5.6	0.2	10.5
Behaves	3.93	3.81	3.0	9.8	0	14.4	2.56	2.85	1.5	7.3	0	10.9
Cares for self	2.54	3.38	1.1	7.5	0	13.8	1.94	2.33	0.8	4.6	0	9.4
Uses hands	1.94	3.05	0.9	5.3	0	13.3	1.06	1.73	0.5	1.8	0	8.8
Understands others	2.42	3.33	0.8	7.0	0	13.6	1.98	2.46	0.6	6.5	0.1	8.7
Focuses attention	3.73	3.8	2.6	9.4	0	13.8	2.03	2.56	0.7	5.7	0.1	12.5
Talks	2.97	3.45	1.6	8.0	0	13.9	3.2	3.58	2.0	8.0	0	14.7
Expresses emotion	3.24	3.56	2.7	8.0	0	13.9	2.84	2.67	2.1	6.64	0	10
Overall rating	2.67	3.35	1.2	7.6	0	13.8	2.75	2.7	1.7	6.9	0.1	9.7

*SD, Standard Deviation; 90th p, 90th percentile; Min, Minimum Score; Max, Maximum Score*.

Correlations between the EYCI and domains of the BSID-III indicated low and non-significant correlations between the EYCI maximum item score and motor functioning domain of the BSID-III for parents (<0.20). We tested the ability of the EYCI to identify borderline delay (i.e., the −1.5SD cut-off on the BSID-III) with 7 children identified as cases; the low number of children who scored below the −2SD threshold (*n* = 1) meant we were unable to test the threshold for probable delay. For borderline delay, agreement for parents was at the high range of moderate (AUC = 0.88 [95% CI: 0.76-1.00]), while that for educators was at the low range of moderate with very wide confidence intervals (AUC = 0.74 [95% CI: 0.56-0.92]).

### Summary of Phase I Results

The AUC results suggest the parent version of the EYCI is able to identify children who might have a delay. Results with educators were less strong. The wide confidence intervals suggest the need to test the tool with a larger sample. Results from both parents and educators' low endorsement of both motor items and low correlations between the motor domain of the BSID-III. We modified the tool to include examples for each motor item to prompt parents and educators to consider motor movements more specifically.

### Phase II: Validation Results

The internal consistency for the parent and educator EYCI remained excellent (alpha = 0.92; 95% CI: 0.91-0.94; alpha = 0.95; 95% CI: 0.94-0.96, respectively). Agreement between parents and educators was generally low (Rho > 0.30). One exception was the *How this child talks* item which demonstrated more moderate agreement (Rho = 0.42, *p* < 0.01). Agreement for the maximum item score was also low (Rho = 0.35, *p* < 0.05).

Both parents and educators showed little domain specificity in agreement between domains on the EYCI and the closest corresponding BSID-III domain. For parents correlations between matched domains on the EYCI and BSID-III were no higher than the EYCI and minimum BSID-III score (see Table 3A). For the educator-completed EYCI, all EYCI items showed the strongest relationship with the language domain of the BSID-III (see Table 3B).

**Table 3:** Associations between the EYCI and BSID-III domains.

	**BSID-III Scores**					
	**Cog**.	**Lang**.	**Mot**.	**Adap**.	**Soc**.	**Min**.
**(A) PARENTS**						
**EYCI Score**						
Get along with others	−0.08	−0.14	−0.05	−0.13	−0.24^**^	−0.19^**^
Moves	−0.13	−0.07	−0.11	−0.23^**^	−0.18^*^	−0.18^*^
Learns	−0.18^*^	−0.26^**^	−0.18^*^	−0.29^**^	−0.35^**^	−0.26^**^
Behaves	−0.13	−0.20^**^	−0.11	−0.22^**^	−0.34^**^	−0.21^**^
Cares for self	−0.11	−0.14^*^	−0.07	−0.17^*^	−0.32^**^	−0.20^**^
Uses hands	−0.16^*^	−0.12	−0.13	−0.28^**^	−0.37^**^	−0.24^**^
Understands others	−0.23^**^	−0.28^**^	−0.16^*^	−0.29^**^	−0.33^**^	−0.28^**^
Focuses attention	−0.20^**^	−0.13	−0.07	−0.22^**^	−0.35^**^	−0.22^**^
Talks	−0.18^*^	−0.34^**^	−0.21^**^	−0.28^**^	−0.33^**^	−0.31^**^
Expresses emotion	−0.13	−0.13	−0.12	−0.31^**^	−0.36^**^	−0.23^**^
Overall	−0.19^**^	−0.26^**^	−0.20^**^	−0.30^**^	−0.35^**^	−0.31^**^
Item maximum	−0.21^**^	−0.29^**^	−0.13	−0.33^**^	−0.38^**^	−0.35^**^
**(B) EDUCATORS**						
Get along with others	−0.05	−0.15^*^	0.00	−0.04	−0.04	−0.08
Moves	−0.08	−0.18^*^	−0.02	0.02	0.09	0.00
Learns	−0.11	−0.30^**^	−0.03	−0.07	−0.02	−0.07
Behaves	−0.05	−0.21^**^	0.01	−0.07	−0.05	−0.07
Cares for self	0.00	−0.14	−0.03	0.01	0.09	−0.07
Uses hands	−0.10	−0.23^**^	−0.02	0.02	0.02	−0.07
Understands others	−0.15^*^	−0.34^**^	−0.04	−0.04	−0.04	−0.16^*^
Focuses attention	−0.10	−0.25^**^	−0.02	−0.03	−0.02	−0.12
Talks	−0.07	−0.40^**^	−0.17^*^	−0.11	−0.11	−0.26^**^
Expresses emotion	−0.08	−0.18^*^	−0.04	−0.07	−0.04	−0.1
Overall	−0.09	−0.37^**^	−0.15^*^	−0.11	−0.16^*^	−0.27^**^
Item maximum	−0.09	−0.30^**^	−0.15^*^	−0.1	−0.12	−0.23^**^

Cog, Cognitive; Lang, Language; Adap, Adaptive; Soc, Social Emotional; Min, Minimum Score.

* p < 0.05;

** *p < 0.01*.

Seven children (3.4 %) in the phase II sample met the −2SD cut-off in the BSID-III and an additional 18 (8.9%) met the −1.5SD cut-off. The parent-completed EYCI performed well for identifying children using the −2SD cut-off, however, results were low to moderate when using the −1.5SD cut-off (see Table 4). Results for educators were poor for both the −2SD (AUC = 0.58 [95% CI: 0.37-0.78]) and the −1.5SD cut-off (AUC = 0.65 [95% CI: 0.54-0.76]).

**Table 4:** Accuracy of the parent completed EYCI.

**BSID-III Cut-off**	**Cases (*n*)**	**AUC**	**CI**	**Threshold**	**Sensitivity**	**Specificity**	**PPV**	**NPV**
-2SD	7	0.85	0.76-0.94	8.65	86%	82%	14.60%	99.40%
−1.5SD	18	0.70	0.59-0.81	4.95	80%	49%	19%	94.70%

*BSID-III, Bayley Scales of Infant and Toddler Development; AUC, Area under the curve; PPV, positive predictive value; NPV, negative predictive power*.

Based on ROC results, a maximum item score of 8.65 on the EYCI can identify children with a probable delay as identified on the BSID-III with good sensitivity and specificity (see Table 4). The positive predictive value for detecting probable delay was low (see Table 4; 6 children correctly identified and 35 false positives). When identifying children who meet the borderline cut-off, an EYCI score of 4.95 provides good sensitivity (but unacceptable specificity. The positive predictive value for identifying borderline functioning was also low (see Table 4).

## Discussion

We developed and tested the EYCI, a tool for early childcare settings to complement existing monitoring and surveillance efforts in the early years. Considering the differences between the parent and educator completed EYCI, we discuss each separately. While further research is needed with larger samples to better determine the effectiveness of the EYCI, current results show promise and that further investigation is warranted for the parent-completed tool. The parent version of the EYCI was able to identify children who scored very low (-2SD) on the BSID-III with reasonable accuracy. The performance of the EYCI in identifying children with milder delays was not as strong. This is consistent with previous research that has reported detecting delay in milder cases is more challenging (10).

While there are no widely accepted guidelines for developmental surveillance, the identified EYCI threshold for identifying children who scored very low on the BSID-III meet recommended guidelines for early developmental screening (16). The sensitivities and specificities of the EYCI are consistent with some of the higher estimates of the PEDS (20, 21). EYCI results with the borderline functioning (-1.5SD) cut-off on the BSID-III were not as strong. While it was possible to identify a value with good sensitivity, specificity is quite low.

Results suggest that the EYCI is likely to over identify children with potential delay. The EYCI's PPV for the very low BSID-III was low. The PPV for the EYCI is lower than prior work on the PEDS (20–23). The low PPV is impacted by the low prevalence (3.4%) of delay in the sample compared to prior work on the PEDS (20–23). While the prevalence of delay in our sample is lower than estimates of delay or disability (16–18), we excluded children with a diagnosed developmental condition. This lowers the prevalence of delay, but provided a more accurate test of the functioning of the tool, as asking about “concerns” is problematic for children with diagnosed delays. This prevalence is consistent with other community-based samples (25).

While this is an important limitation, it is important to note that the purpose of the EYCI is to identify parental concerns that recommend further actions. When parents reach or exceed the threshold for high concerns that was used to identify children very low on the BSID-III (-2SD), actions might include discussion with a professional or completing a screening tool. When parents reach or exceed the threshold for elevated concerns (-1.5SD) monitoring concerns in the short term, or discussion with a professional might be warranted. This is consistent with recommendations for screening in the early years, as the screener would only take place in the presence of parental concerns. As such, the EYCI is ideally used within a supportive relationship with an early-years professional where conversation and discussion can take place to explore parent's concerns.

We propose this approach limits the risks and costs of false positives in a community-based surveillance context. Conversations with professionals can help identify and address the cause of the parental concern even when a delay is not present. This is important as unaddressed parental concerns have been linked to lower scores on developmental tests (though not delayed scores), parental well-being, parenting behaviors, and relationships among family members (53–55). Rather than considering concerns that do not correspond to delay as error, there is evidence that parents engage in a close observation of their children and their health, temperament, and environment, which all play a role in parents' level of concern (56, 57). Research regarding the implications of a false positive, in terms of impacts on families, educators, and services is required to better assess the impact of a false positive test. This work is important as, the PPV for any tool will be low in contexts when prevalence is also low (15).

Phase II results were not as strong at identifying borderline cases of delay as the phase I results, although there were overlapping confidence intervals for the two phases of work. There were no demographic differences in the samples that address this discrepancy. There were some differences in maternal education between the samples, however past research has suggested parent concerns are valid across different levels of parental education (23). One difference between samples that might be relevant was the higher proportion of children identified with a motor delay in phase II compared to phase I. The EYCI showed a low and non-significant correlation with the motor domain of the BSID-III. This suggests the EYCI might not be good at detecting motor delays. Unfortunately, there were too few cases to assess accuracy of the EYCI by BSID-III domain. This highlights the need for further testing in a larger sample to better identify if there are domains that the EYCI might be more limited in addressing.

The weak agreement between the EYCI and BSID on motor functioning is an important area to address in the tool. Problems with the motor items, including low endorsement and low agreement with the BSID-III prompted changes to these items. While the endorsement of motor concerns was more consistent with other EYCI items in phase II than phase I, these changes did not appear to increase the relationship between the EYCI and motor domain of the BSID-III. These results could reflect parents' lack of awareness regarding motor delays.

Educator results in the larger validation sample showed poor agreement with the BSID-III, suggesting that, in the study context, educators were not able to identify developmental problems with sufficient accuracy. The agreement between educators and an independent assessment in the current study were poorer than reported in prior research. The childcare settings included in the current study was broader than prior research, which has focused on LCC centres. While including broader early childcare settings allowed for increased reach, educators in these settings do not have the same opportunities to observe children in these centres as LCC. Children in LCC's attend on a regular basis which allows educators more frequent contact and observations. There is more variability in EarlyOn centres, where some families might attend programs regularly and others infrequently. Further research examining the tools accuracy in different childcare settings can help identify what conditions might be necessary for accurate identification of delay by educations. In particular, it would be useful to determine what minimum level of contact or observation is required to improve accuracy to an acceptable level.

The different patterns of correlations between parent- and educator-completed EYCI with the BSID-III domains suggest parent and educator concerns are distinct. For parents, EYCI items appear to capture general concerns regarding how a child is developing, rather than concerns that are specific to a particular domain of functioning. Past research has reported some distinctions regarding the specificity of parental concerns, with some research indicating specificity regarding the domain of parental concern and functional impairment of the child (58), and other studies indicating non-specific relationships (53). The variability in sampling strategies (general population versus at-risk) and outcomes used (identifying a specific disorder versus any delay) limits the ability to identify clear patterns in the literature. Educators' concerns on the EYCI were almost solely related to language development on the BSID-III. To our knowledge, this is the first study that has examined the specificity of early educator concerns. The difference in how parents and educators form concerns might help explain both the lack of item-level agreement on the EYCI and the large discrepancy in accuracy between parents and educators. The focus on language for educators suggests that training educators on other domains, including cognitive and motor skills, could potentially improve the accuracy of the tool for educators. Considering the poor performance of the educator-completed EYCI in both phases of work at both thresholds of the BSID-III, the EYCI is not recommended for use by educators at this time.

A limitation of the study was the small proportion of children who demonstrated a developmental delay. Considering that delays are not the only source of parental concerns, an important question for future research is to assess the ability of the EYCI to predict future problems that include diagnosis, but also other developmental issues such as school readiness.

## Conclusions

This study was the first test of the validity of the EYCI tool. The EYCI measures parent and educator concerns regarding children with potential developmental delay in the early years. Results suggest promise for the parent-completed EYCI, but further work and testing are needed. Specifically we recommend testing in a larger sample, examining the impact of false positives, and comparing the EYCI to existing practices or tools. The tool is not recommended for educator completion at this time.

## Data Availability Statement

The datasets generated for this study are available on request to the corresponding author.

## Ethics Statement

The studies involving human participants were reviewed and approved by Hamilton Integrated Research Ethics Boards. Written informed consent to participate in this study was provided by the participants' legal guardian.

## Author Contributions

JC, HC, and KN are the principal investigators who designed the study, drafted the manuscript, and made significant revisions to the drafted paper. SV also contributed to the study design and analysis. SV, LR, WC, and CR are co-investigators who contributed to the study design and revisions of the manuscript. All authors read, edited and approved the final manuscript.

## Conflict of Interest

The authors declare that the research was conducted in the absence of any commercial or financial relationships that could be construed as a potential conflict of interest.

## References

[1] Zwaigenbaum L Bauman ML Stone WL Yirmiya N Estes A Hansen RL. Early identification of autism spectrum disorder: recommendations for practice and research. Pediatrics. (2015) 136(4 Suppl. 1):S10–40. 10.1542/peds.2014-3667C 26430168 PMC9923897

[2] Barger B Rice C Simmons CA Wolf R. A systematic review of part c early identification studies. Topics Early Child Spec Educ. (2018) 38:4–16. 10.1177/0271121416678664 28479651 PMC5418588

[3] Rice CE Naarden Braun KV Kogan MD (CDC) C for DC P. Screening for developmental delays among young children-National survey of children's health, united states, (2007). MMWR Suppl. (2014) 63:27–35. 25208255

[4] Hertzman C Clinton J Lynk A Canadian Paediatric Society EYTF. Measuring in support of early childhood development. Paediatr Child Health. (2011) 16:655–60. 10.1093/pch/16.10.655 23204908 PMC3225478

[5] Anderson LM Shinn C Fullilove MT Scrimshaw SC Fielding JE Normand J. The effectiveness of early childhood development programs: a systematic review. Am J Prev Med. (2003) 24:32–46. 10.1016/S0749-3797(02)00655-4 12668197

[6] Berlin LJ Brooks-Gunn J McCarton C McCormick MC. The effectiveness of early intervention: examining risk factors and pathways to enhanced development. In: Feldman MA, editor. Early Intervention. London, UK: Wiley Online Books. (2004). p. 134–50. 10.1002/9780470755778.ch5 9579002

[7] Thomas EM Readiness to Learn at School Among Five-Year-Old Children in Canada. Ottawa, ON: Statistics Canada (2006).

[8] Lonigan CJ Phillips BM Clancy JL Landry SH Swank PR Assel M. Impacts of a comprehensive school readiness curriculum for preschool children at risk for educational difficulties. Child Dev. (2015) 86:1773–93. 10.1111/cdev.12460 26510099

[9] Care CTF on PH Recommendations on screening for developmental delay. Cmaj. (2016) 188:579–87. 10.1503/cmaj.151437 27026672 PMC4868607

[10] Committee BFS Committee MHI for CWSNPA Identifying infants and young children with developmental disorders in the medical home: an algorithm for developmental surveillance and screening. Pediatrics. (2006) 118:405–20. 10.1542/peds.2006-1231 16818591

[11] Siu AL. Screening for speech and language delay and disorders in children aged 5 years or younger: US preventive services task force statement. Pediatrics. (2015) 136:e474–81. 10.1542/peds.2015-1711 26152670

[12] Sand N Silverstein M Glascoe FP Gupta VB Tonniges TP O'Connor KG. Pediatricians' reported practices regarding developmental screening: do guidelines work? Do they help? Pediatrics. (2005) 116:174–9. 10.1542/peds.2004-1809 15995049

[13] Cairney J Clark HJ Nair K Parental concerns, developmental temperature taking, and the necessary conditions for developmental surveillance and screening. Curr Dev Disord Reports. (2016) 3:174–9. 10.1007/s40474-016-0095-5

[14] Glascoe FP. Evidence-based approach to developmental and behavioural surveillance using parents' concerns. Child Care Health Dev. (2000) 26:137–49. 10.1046/j.1365-2214.2000.00173.x 10759753

[15] Streiner DL Issues in screening for developmental delay or disorders. Curr Dev Disord Reports. (2016) 3:180–3. 10.1007/s40474-016-0089-3

[16] Committee on Children With Disabilities Developmental surveillance and screening of infants and young children. Pediatrics. (2001) 108:192–5. 10.1542/peds.108.1.192 11433077

[17] Olusanya BO Davis AC Wertlieb D Boo N-Y Nair MKC Halpern R Developmental disabilities among children younger than 5 years in 195 countries and territories, 1990-2016: a systematic analysis for the global burden of disease study (2016). Lancet Glob Heal. (2018) 6:e1100–21. 10.1016/S2214-109X(18)30309-7 PMC6139259 30172774

[18] Rosenberg SA Zhang D Robinson CC. Prevalence of developmental delays and participation in early intervention services for young children. Pediatrics. (2008) 121:e1503–9. 10.1542/peds.2007-1680 18504295

[19] Glascoe FP The validation and standardization of parents' evaluations of developmental status. Diagnostique. (1999) 23:185–203. 10.1177/073724779802300401

[20] Sices L Stancin T Kirchner L Bauchner H PEDS and aSQ developmental screening tests may not identify the same children. Pediatrics. (2009) 124:4 10.1542/peds.2008-2628 PMC2764374 19736268

[21] Glascoe FP. Parents' evaluation of developmental status: how well do parents' concerns identify children with behavioral and emotional problems? Clin Pediatr (Phila). (2003) 42:133–8. 10.1177/000992280304200206 12659386

[22] Limbos MM Joyce DP. Comparison of the ASQ and PEDS in screening for developmental delay in children presenting for primary care. J Dev Behav Pediatr. (2011) 32:499–511. 10.1097/DBP.0b013e31822552e9 21760526

[23] Glascoe FP Using parents' concerns to detect and address developmental and behavioural problems. J Spec Pediactric Nurs. (1999) 4:1 10.1111/j.1744-6155.1999.tb00077.x 10334009

[24] Squires J Bricker D Potter L Revision of a parent-Completed developmental screening tool: ages and stages questionnaires 1. J Pediatr Psychol. (1997) 22:313–28. 10.1093/jpepsy/22.3.313 9212550

[25] Veldhuizen S Clinton J Rodriguez C Wade TJ Cairney J. Concurrent validity of the ages and stages questionnaires and bayley developmental scales in a general population sample. Acad Pediatr. (2015) 15:231–7. 10.1016/j.acap.2014.08.002 25224137

[26] Velikonja T Edbrooke-Childs J Calderon A Sleed M Brown A Deighton J. The psychometric properties of the ages & stages questionnaires for ages 2-2.5: a systematic review. Child Care Health Dev. (2017) 43:1–17. 10.1111/cch.12397 27554865

[27] Yue A Jiang Q Wang B Abbey C Medina A Shi Y. Concurrent validity of the ages and stages questionnaire and the bayley scales of infant development iII in china. PLoS ONE. (2019) 14:e0221675. 10.1371/journal.pone.0221675 31487302 PMC6728026

[28] Hix-Small H Marks K Squires J Nickel R. Impact of implementing developmental screening at 12 and 24 months in a pediatric practice. Pediatrics. (2007) 120:381–89. 10.1542/peds.2006-3583 17671065

[29] Rydz D Srour M Oskoui M Marget N Shiller M Birnbaum R. Screening for developmental delay in the setting of a community pediatric clinic: a Prospective assessment of parent-Report questionnaires. Pediatrics. (2006) 118:e1178–e86. 10.1542/peds.2006-0466 17015506

[30] Williams R Clinton J Biscaro A. Ontario and the enhanced 18-month well-baby visit: trying new approaches. Paediatr Child Health. (2008) 13:850–6. 10.1093/pch/13.10.850 19436551 PMC2603505

[31] Hack M Taylor HG Drotar D Schluchter M Cartar L Wilson-Costello D. Poor predictive validity of the bayley scales of infant development for cognitive function of extremely low birth weight children at school age. Pediatrics. (2005) 116:333–41. 10.1542/peds.2005-0173 16061586

[32] Murray GK Jones PB Kuh D Richards M. Infant developmental milestones and subsequent cognitive function. Ann Neurol. (2007) 62:128–36. 10.1002/ana.21120 17487877 PMC3465788

[33] Glascoe FP Dworkin PH. The role of parents in the detection of developmental and behavioral problems. Pediatrics. (1995) 95:829–36. 7539122

[34] Hirai AH Kogan MD Kandasamy V Reuland C Bethell C. Prevalence and variation of developmental screening and surveillance in early childhood. JAMA Pediatr. (2018) 172:857–66. 10.1001/jamapediatrics.2018.1524 29987317 PMC6143066

[35] Williams R Clinton J Bennett S Hertzman C Leduc D. Getting it right at 18 months: in support of an enhanced well-baby visit. Paediatr Child Health. (2011) 16:647–50. 10.1093/pch/16.10.647 23204907 PMC3303471

[36] Branson D Vigil DC Bingham A Community childcare providers' role in the early detection of autism spectrum disorders. Early Child Educ J. (2008) 35:523–30. 10.1007/s10643-008-0243-6

[37] Zhang D Krieber-Tomantschger I Poustka L Roeyers H Sigafoos J Bölte S. Identifying atypical development: a Role of day-Care workers? J Autism Dev Disord. (2019) 49:3685–94. 10.1007/s10803-019-04056-3 31144232 PMC6667412

[38] Magdalena J Brinkman SA Duku EK Validity and psychometric properties of the early development instrument in Canada, Australia, United States, and Jamaica. (2011). 103:283–97. 10.1007/s11205-011-9846-1

[39] Thorell LB Nyberg L. The childhood executive functioning inventory (CHEXI): a New rating instrument for parents and teachers. Dev Neuropsychol. (2008) 33:536–52. 10.1080/87565640802101516 18568903

[40] Walker HM Severson HH Systematic Screening for Behavior Disorders. Reston, VA: The Council (1992).

[41] Shahidullah JD Forman SG Norton AM Harris JF Palejwala MH Chaudhuri A Implementation of developmental screening by childcare providers. Infants Young Child. (2020) 33:21–34. 10.1097/IYC.0000000000000158

[42] Boh A Johnson LA Universal screening to promote early identification of developmental delays: exploring childcare providers' beliefs and practices. Early Child Dev Care. (2018) 188:1694–708. 10.1080/03004430.2016.1278369

[43] Dereu M Raymaekers R Warreyn P Schietecatte I Meirsschaut M Roeyers H. Can child care workers contribute to the early detection of autism spectrum disorders? A Comparison Between Screening Instruments with Child Care Workers Versus Parents as Informants. J Autism Dev Disord. (2012) 42:781–96. 10.1007/s10803-011-1307-9 21691866

[44] Larsen K Aasland A Diseth TH. Identification of symptoms of autism spectrum disorders in the second year of life at day-Care centres by day-Care staff: step one in the development of a short observation list. J Autism Dev Disord. (2018) 48:2267–77. 10.1007/s10803-018-3489-x 29423606

[45] Janvier YM Harris JF Coffield CN Louis B Xie M Cidav Z. Screening for autism spectrum disorder in underserved communities: early childcare providers as reporters. Autism. (2015) 20:364–73. 10.1177/1362361315585055 25991845

[46] Sinha M Child Care in Canada. Spotlight on Canadians: Results from the General Social Survey. Ottawa, ON (2014).

[47] Kamerman SB Gatenio-Gabel S Early childhood education and care in the united states: an overview of the current policy picture. Int J Child Care Educ Policy. (2007) 1:23–34. 10.1007/2288-6729-1-1-23

[48] Kamerman SB Early childhood education and care: an overview of developments in the oECD countries. Int J Educ Res. (2000) 33:7–29. 10.1016/S0883-0355(99)00041-5

[49] Ontario Ministry of Education Early Years and Child Care Annual Report, 2018. (2018). Available online at: http://www.edu.gov.on.ca/childcare/EarlyYearsChildCareAnnualReport2018.pdf

[50] Bayley N Bayley scales of infant and toddler development-third edition. San Antonio, TX Harcourt Assess. J Psychoeduc Assess. (2006) 25:180–90. 10.1177/0734282906297199

[51] Streiner DL Norman GR Cairney J Health Measurement Scales : A Practical Guide to Their Development and Use. 5th Ed Oxford: Oxford University Press (2015). 10.1093/med/9780199685219.001.0001

[52] Fischer JE Bachmann LM Jaeschke R. A readers' guide to the interpretation of diagnostic test properties: clinical example of sepsis. Intensive Care Med. (2003) 29:1043–51. 10.1007/s00134-003-1761-8 12734652

[53] Glascoe FP It's not what it seems: the relationship between parents' concerns and children with global delays. Clin Pediatr (Phila). (1994) 33:292–6. 10.1177/000992289403300507 7519535

[54] Glascoe FP Parents' concerns about children's development: prescreening technique or screening test? Pediatrics. (1997) 99:522– 528. 10.1542/peds.99.4.522 9093291

[55] Glascoe FP Macias MM Wegner LM Robertshaw NS. Can a broadband developmental-Behavioral screening test identify children likely to have autism spectrum disorder? Clin Pediatr (Phila). (2007) 46:801–5. 10.1177/0009922807303928 17641122

[56] Marshall J Coulter ML Gorski PA Ewing A Parent recognition and responses to developmental concerns in young children. Infants Young Child. (2016) 29:102–15. 10.1097/IYC.0000000000000056

[57] Karp EA Ibañez L V Warren Z Stone WL. Brief report: what drives parental concerns about their 18-Month-Olds at familial risk for autism spectrum disorder? J Autism Dev Disord. (2017) 47:1535–41. 10.1007/s10803-017-3060-1 28236100

[58] Chen IC Lee HC Yeh GC Lai CH Chen S-C. The relationship between parental concerns and professional assessment in developmental delay in infants and children–a hospital-based study. J Chinese Med Assoc. (2004) 67:239–44. 15357111

